# Supplementary material for: Proteome Profiling of Brain Vessels in a Mouse Model of Cerebrovascular Pathology
Source: Biology (Basel). 2023 Dec 7;12(12):1500. doi: 10.3390/biology12121500 (PMC10740654; doi:10.3390/biology12121500)
Supplement: Supplementary file 1 [file biology-12-01500-s001.zip › biology-2699701-supplementary.pdf]

Supplementary Material

Cerebrovascular Proteomics Workflow

Protein Extraction from Cerebral Arteries

Briefly, 100 μL of 0.2% (*w*/*v*) Rapigest SF (Waters, Milford, MA, USA) in 50 mM ammonium bicarbonate was added to each of the three biological replicates and subjected to three, short pulses (20 sec each) of sonication for protein solubilization using a probe sonicator (550 Sonic Dismembrator; Fisher Scientific, Ottawa, ON, Canada), followed by incubation (95 °C dry bath, 10 min), and centrifugation (20 min, 10,000× *g*). The supernatant (S0) containing Rapigest SF-solubilized proteins was transferred to a new tube, and 20 μL removed for (a) total protein content analyses, and (b) to run a 1D, 12% SDS-PAGE gel. Conversely, the pellet was resuspended in 50 μL of buffered collagenase (0.1 mg/mL crude collagenase, 50 mM Tris, pH 7.5, 2 mM CaCl2) and incubated (60 min at 37 °C) to further solubilize extracellular matrix and membrane proteins. Thereafter, 80 μL of sample-specific S0 was added to the above mix, and the entire mixture sonicated three times (30 s each), followed by incubation (10 min, 95 °C), and centrifugation (20 min, 10,000× g). The final supernatant (S1) containing solubilized cerebral arterial proteins was collected.

Protein Digest

Proteins in each S1 supernatant were denatured in 100 mM 2-mercaptoethanol (10 min, 95 °C). Following a 2 min cool down at room temperature (RT), 6 μL of N-Glycosidase F

(Roche, Cat. # 11365185001) was added to each sample and incubated overnight at 37 °C to deglycosylate glycoproteins, which increases detection of membrane and secreted proteins by mass spectrometry [1]. Thereafter, each sample was reduced in fresh 4 mM dithiothreitol (10 min at 95 °C), and then alkylated in the dark in 10 mM freshly prepared iodoacetamide (30 min at RT). Subsequently, each sample was diluted to 450 μL with Milli-Q water and 5 μL of trypsin enzyme stock solution (Trypsin Gold, mass spectrometry grade, Promega, Cat. # V5280) added, and then incubated overnight (37 °C).

Maximizing Proteome Coverage

As described in detail previously [1,2], we used gel-free proteomics to maximize cerebral arterial proteome coverage. Specifically, tryptic-digest of each S1 protein mix was followed by fractionation of peptides by strong cation exchange [3], and eluted peptide fractions collected from low to high salt-containing cation exchange elution buffer concentration, by slowly injecting (~1 drop/s) 0.5 mL of each elute buffer. Specifically, peptide fractions were collected at 20%, 40% and 100% elution buffer concentrations [3].

Mass Spectrometry Analysis

For all 18 samples, each strong cation exchange fraction was analyzed by nanoLC-MS/MS, culminating in a grand total of 162 runs. Specifically, each fraction was analyzed using ESI-LTQ-Orbitrap-XL mass spectrometer (Thermo) coupled to a NanoAcquity UPLC system (Waters, Milford, MA), as previously described [1,2].

Protein Identification and Bioinformatics

The raw data was converted to mzXML and MGF (Mascot generic format) format files using Msconvert (https://proteowizard.sourceforge.io/). The resulting MS/MS (tandem mass spectrometry) spectra were searched against Mus musculus Swiss-Prot database using Mascot v2.2.0 search engine [4] for protein identification with the following parameters: enzyme = trypsin; modifications = C (carbamidomethyl, fixed), M (oxidation, variable); peptide tolerance = 1.5 Da; fragment tolerance = 1.5 Da; one missed cleavage allowed). Decoy database searches were used to estimate the false positive identification rate of peptides. Peptides with a delta mass >15 ppm and score < 35 were removed and this yielded a false positive identification rate of 1%. In addition, Peptide Prophet probabilities, an independent statistical measure of peptide identification, yielded $p \geq 0.95$ for all identified peptides. To extract quantitative MS (mass spectrometry) data, to align all runs and integrate protein search results, in-house software MatchRx 2 version QnD-2.0 was used. Peptide intensity validation was performed using MSight software version 1.0 (http://www.expasy.ch/MSight). The intensities of the peptides in each sample were normalized using median normalization to correct for run-to-run variability and possible unequal injections of the samples. Only peptides that were matched among all the samples were used for normalization. Median intensity of the matched peptides for each run (Mi) and for all the runs (Ma) were calculated and the intensity of each peptide (matched and unmatched) was normalized as follows: Normalized intensity = (Original

intensity) × Ma/Mi.

Supplemental Figures

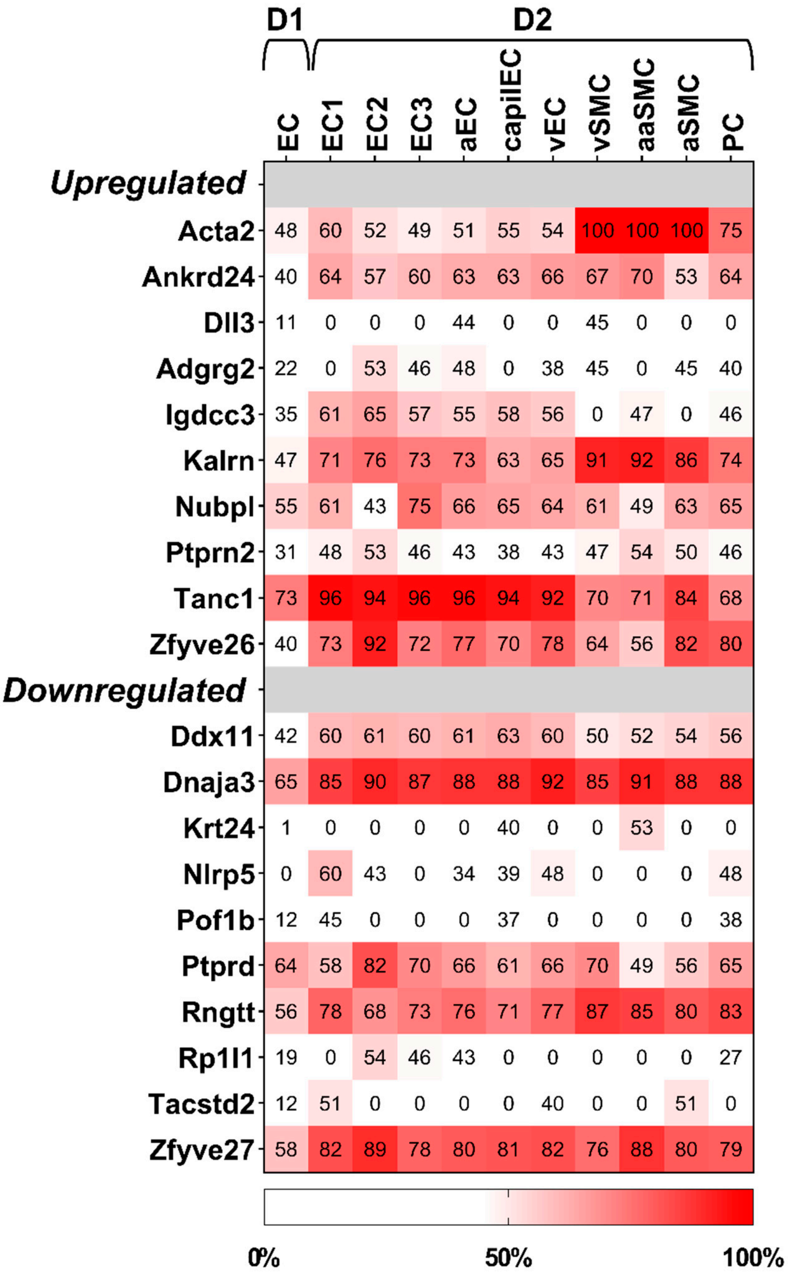

Supplemental Figure S1. Relative gene expression of List 2 proteins in public vascular datasets. Relative gene expression of the 20 List 2 proteins in mouse cerebrovascular

single cell transcriptome databases D1 [5] and D2 [6]. Abbreviations: EC, endothelial cell; aEC, arterial EC; capilEC, capillary EC; vEC, venular EC; vSMC, venular smooth muscle cell; aaSMC, arteriole SMC; aSMC, arterial SMC; PC, pericyte. This figure is duplicated from Figure 4 except raw values are on the heat map.

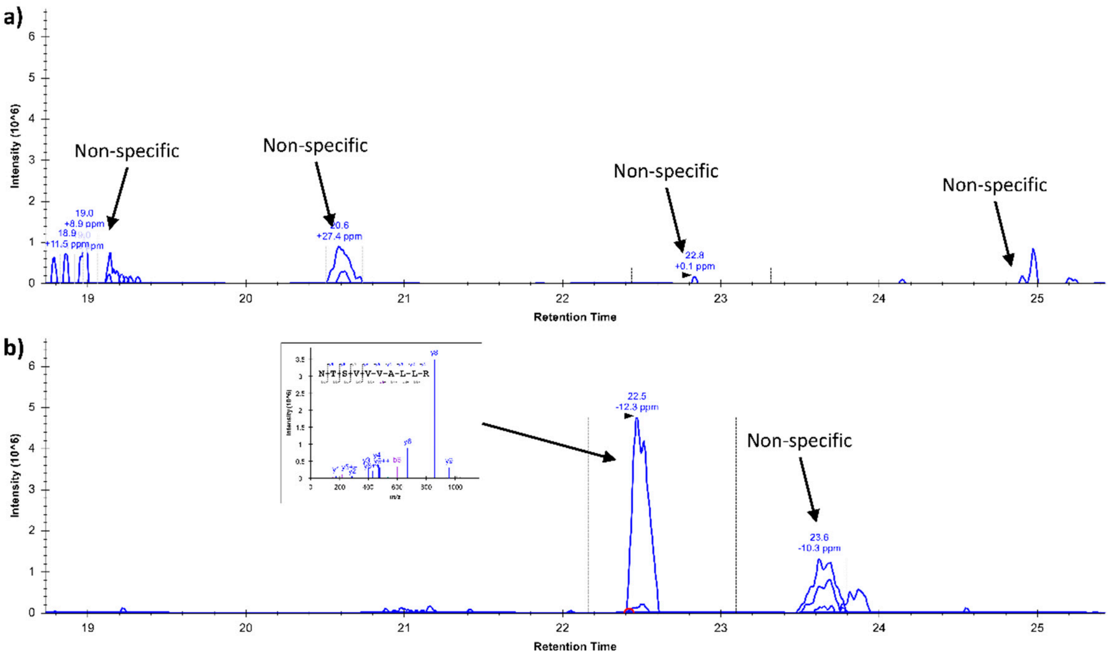

Supplemental Figure S2. Examples of extracted ion-chromatograms and MS/MS spectra in precleared plasma and depleted plasma. (**a**,**b**) An example of extracted ion-chromatogram for a TANC1 peptide in non-depleted (**a**) and depleted (**b**) plasma. Arrows indicate either non-specific peaks or specific peak. TANC1-specific peak was only detected in depleted plasma (**b**) as confirmed by its MS/MS spectra.

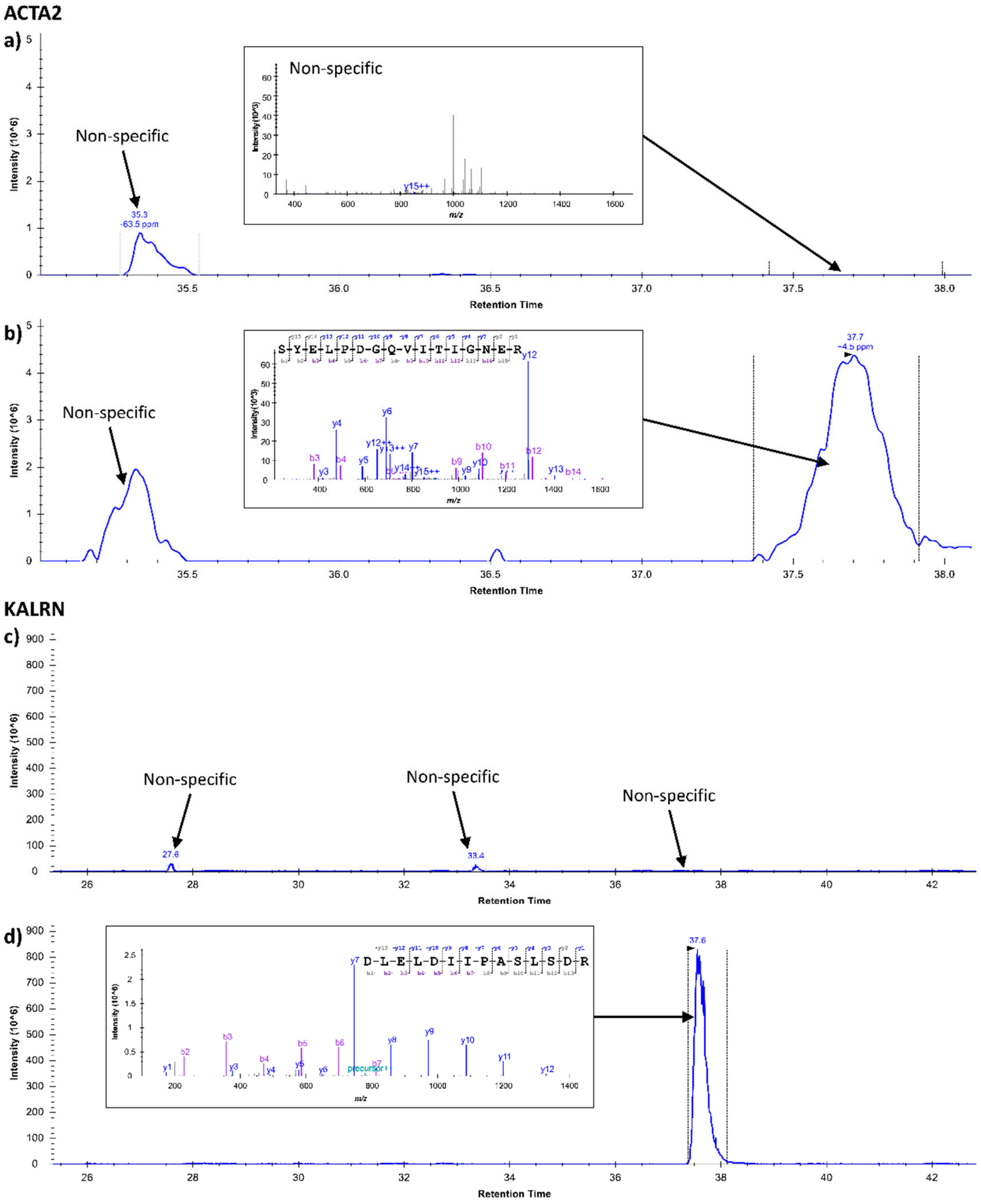

Supplemental Figure S3. Examples of extracted ion-chromatograms and MS/MS spectra in precleared plasma and total plasma EVs. (**a**,**b**) An example of extracted ion-chromatogram for an ACTA2 peptide in non-depleted plasma (**a**) and total EVs (**b**). Arrows indicate either non-specific peaks or specific peak. ACTA2-specific peak was only

detected in total EVs (**b**) as confirmed by its MS/MS spectra. (**c**,**d**) An example of extracted ion-chromatogram for an KALRN peptide in non-depleted plasma (**c**) and total EVs (**d**). Arrows indicate either non-specific peaks or specific peak. KALRN-specific peak was only detected in total EVs (**d**) as confirmed by its MS/MS spectra.

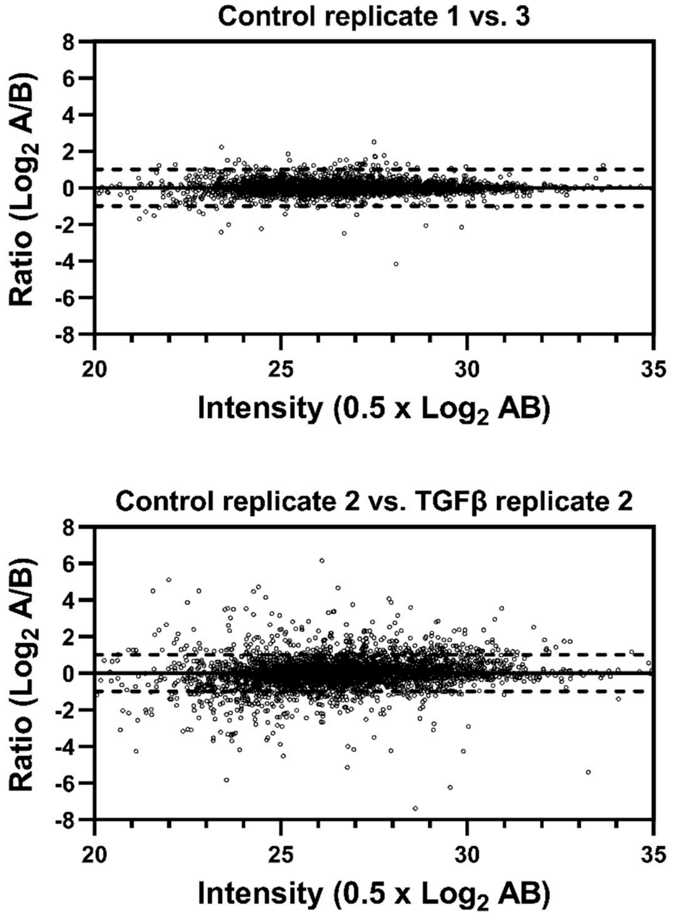

Supplemental Figure S4. The figure shows the reproducibility between two biological runs of control animals (**top panel**) and between control and TGFbeta runs (**bottom panel**). The median relative standard deviation among replicates of control and TGFbeta is 10.1% and 15.7%, respectively and between control and TGFbeta samples is 26%.

Supplemental Tables

Supplementary Table S1. List 1 and List 2 proteins *

Uniprot (ID)	Gene	Gene (Synonyms)	Protein Name (Recommended)	Log2 fold Change	EVs			
					M		H	
					p	r	p	r
Q3UHJ0	Aak1	Kiaa1048	AP2-associated protein kinase 1	−1.09	X		X	X
P62737	Acta2	Actsa, Actvs	Actin, aortic smooth muscle	1.05	X		X	X
Q8CJ12	Adgrg2	Gpr64, Me6	Adhesion G-protein coupled receptor G2	4.69	X		X	X
Q80VM7	Ankrd24	-	Ankyrin repeat domain-containing protein 24	1.08			X	
Q8BVM2	Antxrl	-	Anthrax toxin receptor-like	−1.61				
O88879	Apaf1	-	Apoptotic protease-activating factor 1	−1.33	X		X	X
Q9Z1K5	Arih1	Ari, Ubch7bp	E3 ubiquitin-protein ligase ARIH1	−1.3			X	X
Q80VC9	Camsap3	Kiaa1543	Calmodulin-regulated spectrin-associated protein 3	−2.18				X
Q80ZU5	Ccdc181	-	Uncharacterized Coiled-coil domain-containing protein 181 protein C1orf114 homolog	1.33				
P06909	Cfh	Hf1	Complement factor H	1.14	X		X	X
Q99388	Csprs	D1Lub1	Component of Sp100-rs	−2.05		X		
Q02248	Ctnnb1	Catnb	Catenin beta-1	−1.32	X		X	X
A2AKB9	Dcaf10	Wdr32	DDB1- and CUL4-associated factor 10	−1.25				X
Q91VR5	Ddx1	-	ATP-dependent RNA helicase DDX1	1.11	X		X	X
Q6AXC6	Ddx11	-	Probable ATP-dependent RNA helicase DDX11	−1.08			X	X
Q9CWT6	Ddx28	-	Probable ATP-dependent RNA helicase DDX28	1.35			X	X
Q9R0Z9	Dlc1	Arhgap7, Stard12	Rho GTPase-activating protein 7	1.71			X	X
Q811D0	Dlg1	Dlgh1	Disks large homolog 1	−1.46	X		X	X
O88516	Dll3	-	Delta-like protein 3	1.48			X	X
Q99M87	Dnaja3	Tid1	DnaJ homolog subfamily A member 3, mitochondrial	−1.95	X		X	X
Q8BVG4	Dpp9	-	Dipeptidyl peptidase 9	1.62	X		X	X
O70251	Eef1b	Eef1b2	Elongation factor 1-beta	1.51	X		X	X
O08810	Eftud2	Snrp116	116 kDa U5 small nuclear ribonucleoprotein component	−2.28	X		X	X
Q6DYE8	Enpp3	-	Ectonucleotide pyrophosphatase/phosphodiesterase family member 3	1.01	X		X	
Q9D281	Fam114a1	Noxp20	Protein Noxp20	−1.54			X	
Q8K285	Fcho1	-	F-BAR domain only protein 1	−1.65			X	X
Q8CG64	Fkrp	-	Ribitol 5-phosphate transferase FKRP	1.01	X			X
Q7TQ32	Hjv	Hfe2, Rgmc	Hemojuvelin	−1.84				
Q8R0J8	Idnk	-	Probable gluconokinase	1.03			X	X
Q8BQC3	Igdcc3	Punc	Immunoglobulin superfamily DCC subclass member 3	1.46				X
Q920Q8	Ivns1abp	Kiaa0850, Nd1, Nd1L,	Influenza virus NS1A-binding protein homolog	−1.55				X

		Nd1S, Ns1, Ns1bp						
A2CG49	**Kalrn**	-	Kalirin	1.02			X	X
Q03719	**Kcnd1**	-	Potassium voltage-gated channel subfamily D member 1	−1.75			X	X
P28740	**Kif2a**	Kif2, Kns2	Kinesin-like protein KIF2A	−1.12	X		X	X
Q8VCK5	**Klhl20**	Kiaa4210, Kleip	Kelch-like protein 20	−1.15			X	X
A1L317	**Krt24**	Ka24	Keratin, type I cytoskeletal 24	−1.02	X		X	
Q0P5X1	**Lrriq1**	-	Leucine-rich repeat and IQ domain-containing protein 1	−1.14	X		X	
Q9QYR6	**Map1a**	Mtap1, Mtap1a	Microtubule-associated protein 1A	−1.74			X	X
Q9WUU9	**Mcm3ap**	Ganp, Map80	Germinal-center associated nuclear protein	−1.06			X	X
Q6PDC8	**Mfsd4**		Major facilitator superfamily domain-containing protein 4	1.78			X	
P97479	**Myo7a**	Myo7	Unconventional myosin-VIIa	−1.51	X		X	
Q9D5Y0	**na**	-	Uncharacterized protein C7orf31 homolog	1.39				
Q9CQH3	**Ndufb5**	-	NADH dehydrogenase [ubiquinone] 1 beta subcomplex subunit 5, mitochondrial	−1.4	X		X	X
Q9R1M5	**Nlrp5**	Mater, Nalp5	NACHT, LRR and PYD domains-containing protein 5	−1.05				
Q9CWD8	**Nubpl**	-	Iron-sulfur protein NUBPL	2.39			X	X
Q6P3D0	**Nudt16**	-	U8 snoRNA-decapping enzyme	−1.32			X	X
Q61036	**Pak3**	Pak-3, Pakb, Stk4	Serine/threonine-protein kinase PAK 3	−2.25	X		X	
P61249	**Pde6h**	-	Retinal cone rhodopsin-sensitive cGMP 3',5'-cyclic phosphodiesterase subunit gamma	−1.51				
Q8BUY9	**Pggt1b**	-	Geranylgeranyl transferase type-1 subunit beta	−1.07			X	X
P26450	**Pik3r1**	-	Phosphatidylinositol 3-kinase regulatory subunit alpha	−1.09			X	X
Q8K4L4	**Pof1b**	-	Protein POF1B	−1.76			X	
Q8R4S0	**Ppp1r14c**	Kepi	Protein phosphatase 1 regulatory subunit 14C	1.27				X
Q8BQ30	**Ppp1r18**	-	Phostensin	1.17		X	X	X
Q0VGB7	**Ppp4r2**	-	Serine/threonine-protein phosphatase 4 regulatory subunit 2	−1.07			X	X
Q91WG5	**Prkag2**	-	5'-AMP-activated protein kinase subunit gamma-2	1.2	X		X	X
Q64487	**Ptprd**		Receptor-type tyrosine-protein phosphatase delta	−1.45	X		X	
P80560	**Ptprn2**	-	Receptor-type tyrosine-protein phosphatase N2	1.23				X
O55236	**Rngtt**	Cap1a	mRNA-capping enzyme	−1.41			X	X
Q8CGM2	**Rp1l1**	Rp1hl1	Retinitis pigmentosa 1-like 1 protein	−1.34				
O70258	**Sgce**	-	Epsilon-sarcoglycan	−1.97	X		X	X
Q80W37	**Snupn**	Rnut1	Snurportin-1	−1.02			X	X
Q505B8	**Syce2**	Cesc1	Synaptonemal complex central element protein 2	1.07				
Q9CUU3	**Sycp2**	Scp2	Synaptonemal complex protein 2	−1.21	X		X	

Q8BGV3	Tacstd2	Trop2	Tumor-associated calcium signal transducer 2	−1.31			X	
Q0VGY8	Tanc1	-	Protein TANC1	1.11	X		X	X
Q7TQA6	Tas2r38	T2r31, Tas2r138	Taste receptor type 2 member 38	1.31				
P70325	Tbx4	-	T-box transcription factor TBX4	1.63				
A8C756	Thada	Kiaa1767	Thyroid adenoma-associated protein homolog	2.62			X	X
P39447	Tjp1	Zo1	Tight junction protein ZO-1	−1.12	X		X	X
Q6A070	Togaram1	Fam179b, Kiaa0423	TOG array regulator of axonemal microtubules protein 1	1.39				
Q9CYZ2	Tpd52l2	-	Tumor protein D54	−1.09	X		X	X
Q80VC6	Trnau1ap	Secp43, Trspap1	tRNA selenocysteine 1-associated protein 1	1.53				X
Q8VCH8	Ubxn4	Ubxd2, Ubxdc1	UBX domain-containing protein 4	2.33			X	X
Q9JKB1	Uchl3	-	Ubiquitin carboxyl-terminal hydrolase isozyme L3	−2.1	X		X	X
Q91YD6	Vill	Villp	Villin-like protein	−1.11			X	X
Q8BYK8	Zc3h6	Zc3hdc6	Zinc finger CCCH domain-containing protein 6	−1.12			X	
Q5DU37	Zfyve26	Kiaa0321	Zinc finger FYVE domain-containing protein 26	1.13			X	X
Q3TXX3	Zfyve27	-	Protrudin	−1.56			X	X

* List 2 proteins are highlighted in yellow. Note that the Vesiclepedia database, was used to identify List 1 and 2 proteins detected in the TGF cerebrovasculature, and also detected in human extracellular vesicles. Abbreviations: na, not available; EVs, extracellular vesicles; M, mouse; H, human; p, protein; r, mRNA.

Supplementary Table S2. EV markers detected *

**Accession, Protein**	**Symbol**	**Undepleted Plasma**	**Total Plasma EV**
P60033, CD81 antigen	CD81	Not detected	Detected
P21926, CD9 antigen	CD9	Not detected	Detected
Q8WUM4, Programmed cell death 6-interacting protein	PDCD6IP	Not detected	Detected
P01023, Alpha-2-macroglobulin	A2M	Detected	Detected
P60709, Actin, cytoplasmic 1	ACTB	Not detected	Detected
P63261, Actin, cytoplasmic 2	ACTG1	Detected	Detected
P02768, Albumin	ALB	Detected	Detected but significantly depleted
P04075, Fructose-bisphosphate aldolase A	ALDOA	Detected	Detected
P50995, Annexin A11	ANXA11	Not detected	Detected
P07355, Annexin A2	ANXA2	Not detected	Detected
P84077, ADP-ribosylation factor 1	ARF1	Not detected	Detected

P05023, Sodium/potassium-transporting ATPase subunit alpha-1	ATP1A1	Not detected	Detected
P78371, T-complex protein 1 subunit beta	CCT2	Not detected	Detected
P60953, Cell division control protein 42 homolog	CDC42	Not detected	Detected
P23528, Cofilin-1	CFL1	Detected	Detected
O00299, Chloride intracellular channel protein 1	CLIC1	Not detected	Detected
Q00610, Clathrin heavy chain 1	CLTC	Not detected	Detected
P06733, Alpha-enolase	ENO1	Not detected	Detected
P15311, Ezrin	EZR	Not detected	Detected
P21333, Filamin-A	FLNA	Not detected	Detected
O75955, Flotillin-1	FLOT1	Not detected	Detected
P04406, Glyceraldehyde-3-phosphate dehydrogenase	GAPDH	Detected	Detected
P50395, Rab GDP dissociation inhibitor beta	GDI2	Not detected	Detected
P04899, Guanine nucleotide-binding protein G(i) subunit alpha-2	GNAI2	Not detected	Detected
P62873, Guanine nucleotide-binding protein G(I)/G(S)/G(T) subunit beta-1	GNB1	Not detected	Detected
P62879, Guanine nucleotide-binding protein G(I)/G(S)/G(T) subunit beta-2	GNB2	Not detected	Detected
P07900, Heat shock protein HSP 90-alpha	HSP90AA1	Not detected	Detected
P11021, Endoplasmic reticulum chaperone BiP	HSPA5	Not detected	Detected
P11142, Heat shock cognate 71 kDa protein	HSPA8	Detected	Detected
P23229, Integrin alpha-6	ITGA6	Detected	Detected
P05556, Integrin beta-1	ITGB1	Not detected	Detected
P00338, L-lactate dehydrogenase A chain	LDHA	Detected	Detected
P07195, L-lactate dehydrogenase B chain	LDHB	Detected	Detected
Q08380, Galectin-3-binding protein	LGALS3BP	Detected	Detected
Q08431, Lactadherin	MFGE8	Not detected	Detected
P26038, Moesin	MSN	Not detected	Detected
Q14764, Major vault protein	MVP	Not detected	Detected
P35579, Myosin-9	MYH9	Not detected	Detected
P07737, Profilin-1	PFN1	Detected	Detected
P00558, Phosphoglycerate kinase 1	PGK1	Not detected	Detected
P14618, Pyruvate kinase PKM	PKM	Detected	Detected
P62937, Peptidyl-prolyl cis-trans isomerase A	PPIA	Not detected	Detected
Q06830, Peroxiredoxin-1	PRDX1	Not detected	Detected
P32119, Peroxiredoxin-2	PRDX2	Not detected	Detected
P62820, Ras-related protein Rab-1A	RAB1A	Not detected	Detected
P51148, Ras-related protein Rab-5C	RAB5C	Not detected	Detected
P51149, Ras-related protein Rab-7a	RAB7A	Not detected	Detected
P61006, Ras-related protein Rab-8A	RAB8A	Not detected	Detected
P61224, Ras-related protein Rap-1b	RAP1B	Detected	Detected
P61586, Transforming protein RhoA	RHOA	Not detected	Detected
O00560, Syntenin-1	SDCBP	Not detected	Detected
P08195, 4F2 cell-surface antigen heavy chain	SLC3A2	Not detected	Detected
P27105, Stomatin	STOM	Not detected	Detected
P02786, Transferrin receptor protein 1	TFRC	Detected	Detected
P07996, Thrombospondin-1	THBS1	Detected	Detected
P60174, Triosephosphate isomerase	TPI1	Detected	Detected
Q71U36, Tubulin alpha-1A chain	TUBA1A	Not detected	Detected
P68363, Tubulin alpha-1B chain	TUBA1B	Not detected	Detected
Q9BQE3, Tubulin alpha-1C chain	TUBA1C	Not detected	Detected
P22314, Ubiquitin-like modifier-activating enzyme 1	UBA1	Not detected	Detected

P55072, Transitional endoplasmic reticulum ATPase	VCP	Not detected	Detected
P61981, 14-3-3 protein gamma	YWHAG	Not detected	Detected
Q04917, 14-3-3 protein eta	YWHAH	Not detected	Detected
P63104, 14-3-3 protein zeta/delta	YWHAZ	Detected	Detected

* Shown are known proteins from the "Top 100 EV Proteins", defined as proteins that are often identified in EVs (Vesiclepedia database) that were either detected in undepleted plasma and/or total plasma EV isolates by proteomics. Note that albumin is a negative marker for plasma EVs and was significantly depleted (>95%) in total plasma EV compared to the undepleted plasma. Additional MISEV guidelines [7] were also followed, including nanoparticle tracking analysis, to demonstrate the presence of EVs (Star et al., *in preparation*).

Supplementary Table S3. Molecular Functions and Biological Processes of List 2 Proteins in Mice and Human *.

(a)

	ACTA2		ANKRD24		DLL3		ADGRG2		IGDCC3		KALRN		NUBPL		PTPRN2		TANC1		ZFYVE26	
	**Human**	**Mice**	**Human**	**Mice**	**Human**	**Mice**	**Human**	**Mice**	**Human**	**Mice**	**Human**	**Mice**	**Human**	**Mice**	**Human**	**Mice**	**Human**	**Mice**	**Human**	**Mice**
**MOLECULAR FUNCTIONS**	Y	Y	Y	Y	Y	Y	Y	Y			Y	Y	Y	Y	Y	Y			Y	Y
dna binding																				
rna binding																				
catalytic activity																				
gtpase activity											Y	Y								
structural molecule activity																				
cytoskeletal protein binding			Y	Y																
lipid binding																			Y	Y
transferase activity											Y	Y								
hydrolase activity	Y	Y													Y	Y				
molecular transducer activity							Y	Y							Y					
molecular function regulator activity											Y	Y								
catalytic activity, acting on a protein											Y	Y			Y	Y				
catalytic activity, acting on dna																				
catalytic activity, acting on rna																				
molecular carrier activity													Y	Y						
transcription regulator activity																				
atp-dependent activity													Y	Y						
other molecular function	Y	Y			Y	Y					Y	Y	Y	Y					Y	Y
**BIOLOGICAL PROCESSES**	Y	Y	Y	Y	Y	Y	Y	Y	Y	Y	Y	Y	Y	Y	Y	Y	Y	Y	Y	Y
mitotic cell cycle																			Y	Y
cytokinesis																			Y	Y
immune system process																				
muscle system process	Y	Y																		
circulatory system process	Y	Y																		
dna replication																				
dna repair																			Y	Y
dna recombination																			Y	Y
chromatin organization																				
regulation of dna-templated transcription																				
protein folding																				
lipid metabolic process															Y	Y				
sulfur compound metabolic process													Y	Y						
autophagy																			Y	Y
inflammatory response																				
mitochondrion organization													Y	Y						
cytoskeleton organization																				
lysosome organization																			Y	Y
cell adhesion									Y	Y										
programmed cell death																				
mrna metabolic process																				
vesicle-mediated transport											Y									
reproductive process							Y					Y								
signaling	Y	Y			Y	Y	Y	Y			Y	Y			Y	Y				
cell differentiation	Y	Y	Y	Y	Y	Y					Y	Y					Y	Y		
cell junction organization												Y					Y	Y		
cilium organization																				
anatomical structure development	Y	Y	Y	Y	Y	Y					Y	Y					Y	Y		
cell motility	Y	Y																		
nervous system process			Y	Y					Y	Y		Y					Y	Y		
membrane organization																				
protein-containing complex assembly													Y	Y						
protein localization to plasma membrane																				
defense response to other organism																				
other biological process	Y	Y									Y	Y	Y	Y	Y	Y				

(b)

	DDX11		DNAJA3		KRT24		NLRP5		POF1B		PTPRD		RNGTT		RP1L1		TACSTD2		ZFYVE27	
	Human	Mice	Human	Mice	Human	Mice	Human	Mice	Human	Mice	Human	Mice	Human	Mice	Human	Mice	Human	Mice	Human	Mice
**MOLECULAR FUNCTIONS**	Y	Y	Y	Y	Y	Y	Y	Y	Y	Y	Y	Y	Y	Y					Y	Y
dna binding	Y	Y																		
rna binding	Y	Y																		
catalytic activity	Y	Y																		
gtpase activity			Y	Y																
structural molecule activity					Y	Y														
cytoskeletal protein binding							Y	Y	Y	Y										
lipid binding																				
transferase activity			Y										Y	Y						
hydrolase activity	Y	Y	Y								Y	Y	Y	Y						
molecular transducer activity											Y									
molecular function regulator activity			Y	Y																
catalytic activity, acting on a protein			Y								Y	Y	Y	Y						
catalytic activity, acting on dna	Y	Y																		
catalytic activity, acting on rna													Y	Y						
molecular carrier activity																				
transcription regulator activity			Y																	
atp-dependent activity	Y	Y																		
other molecular function	Y	Y	Y	Y			Y	Y			Y	Y	Y	Y					Y	Y
**BIOLOGICAL PROCESSES**	Y	Y	Y	Y	Y	Y	Y	Y	Y	Y	Y	Y	Y	Y	Y	Y	Y	Y	Y	Y
mitotic cell cycle																				
cytokinesis																				
immune system process			Y	Y							Y	Y								
muscle system process																				
circulatory system process																				
dna replication	Y	Y	Y	Y																
dna repair	Y	Y																		
dna recombination																				
chromatin organization	Y	Y																		
regulation of dna-templated transcription	Y	Y	Y	Y																
protein folding			Y	Y																
lipid metabolic process																				
sulfur compound metabolic process																				
autophagy																				
inflammatory response							Y	Y												
mitochondrion organization			Y	Y																
cytoskeleton organization					Y	Y	Y	Y	Y	Y					Y	Y	Y	Y		
lysosome organization																				
cell adhesion			Y	Y							Y	Y					Y	Y		
programmed cell death			Y	Y																
mrna metabolic process													Y	Y						
vesicle-mediated transport							Y	Y											Y	Y
reproductive process							Y	Y												
signaling			Y	Y							Y	Y			Y	Y			Y	Y
cell differentiation			Y	Y	Y	Y			Y	Y	Y	Y			Y	Y	Y	Y	Y	Y
cell junction organization			Y	Y					Y	Y	Y	Y								
cilium organization															Y	Y				
anatomical structure development			Y	Y	Y	Y	Y	Y	Y	Y	Y	Y			Y	Y	Y	Y	Y	Y
cell motility																	Y	Y		
nervous system process															Y	Y	Y			
membrane organization			Y	Y							Y	Y								
protein-containing complex assembly								Y												
protein localization to plasma membrane																			Y	Y
defense response to other organism			Y	Y																
other biological process	Y	Y	Y	Y			Y	Y			Y	Y	Y	Y	Y	Y	Y	Y	Y	Y

* Shown are the known molecular functions and biological processes of the List 2 proteins (from Uniprot database) in mice and human. We have shown molecular functions and biological processes for (a) upregulated and (b) downregulated proteins in TGF mice.

References

1. Badhwar, A.; Stanimirovic, D.B.; Hamel, E.; Haqqani, A.S. The Proteome of Mouse Cerebral Arteries. *J. Cereb. Blood Flow. Metab.* **2014**, *34*, 1033–1046.
2. Badhwar, A.; Brown, R.; Stanimirovic, D.B.; Haqqani, A.S.; Hamel, E. Proteomic Differences in Brain Vessels of Alzheimer's Disease Mice: Normalization by PPARγ Agonist Pioglitazone. *J. Cereb. Blood Flow Metab.* **2016**. https://doi.org/10.1177/0271678X16655172.
3. Haqqani, A.S.; Kelly, J.F.; Stanimirovic, D.B. Quantitative Protein Profiling by Mass Spectrometry Using Label-Free Proteomics. *Methods Mol. Biol.* **2008**, *439*, 241–256.
4. Hirosawa, M.; Hoshida, M.; Ishikawa, M.; Toya, T. MASCOT: Multiple Alignment System for Protein Sequences Based on Three-Way Dynamic Programming. *Comput. Appl. Biosci.* **1993**, *9*, 161–167.
5. Zhang, Y.; Sloan, C.K.; Bennett, S.A.; Scholze, M.L.; Keeffe, O. An RNA-Sequencing Transcriptome and Splicing Database of Glia, Neurons, and Vascular Cells of the Cerebral Cortex. *J. Neurosci.* **2014**, *34*, 11929–11947.
6. Vanlandewijck, M.; He, L.; Mäe, M.A.; Andrae, J.; Ando, K.; Gaudio, D. A Molecular Atlas of Cell Types and Zonation in the Brain Vasculature. *Nature* **2018**, *554*, 475–480.
7. Théry, C.; Witwer, K.W.; Aikawa, E.; Alcaraz, M.J.; Anderson, J.D.; Andriantsitohaina, R.; Antoniou, A.; Arab, T.; Archer, F.; Atkin-Smith, G.K.; et al. Minimal Information for Studies of Extracellular Vesicles 2018 (MISEV2018): A Position Statement of the International Society for Extracellular Vesicles and Update of the MISEV2014 Guidelines. *J. Extracell. Vesicles* **2018**, *7*. https://doi.org/10.1080/20013078.2018.1535750.
